# Short-term traffic-related gaseous pollutant exposure is associated with childhood bronchopneumonia hospitalization: a 10-year time-series study from Guangzhou, China

**DOI:** 10.3389/fpubh.2026.1885590

**Published:** 2026-07-16

**Authors:** Qiuzheng Yue, Qian Nie, Weiling Liu, Guiying Zhuang, Shiqi Yang, Weiqi Liu, Sitao Li, Chuanrui Zhu

**Affiliations:** 1 Division of Neonatology, The Maternal and Children Health Care Hospital (Huzhong Hospital) of Huadu, Guangzhou, Guangdong, China; 2 College of Life Science, South China Agricultural University, Guangzhou, Guangdong, China; 3 Department of Clinical Laboratory, Foshan Fosun Chancheng Hospital, Foshan, Guangdong, China; 4 Department of Pediatrics, The Sixth Affiliated Hospital, Sun Yat-sen University, Guangzhou, Guangdong, China; 5 Biomedical Innovation Center, The Sixth Affiliated Hospital, Sun Yat-sen University, Guangzhou, Guangdong, China; 6 Department of Clinical Laboratory, The Maternal and Children Health Care Hospital (Huzhong Hospital) of Huadu, Guangzhou, Guangdong, China; 7 NICU (Neonatal Intensive Care Unit), Shenzhen Futian District Maternity and Child Healthcare Hospital, Shenzhen, Guangdong, China

**Keywords:** air pollution, bronchopneumonia, child, nitrogen dioxide, sulfur dioxide

## Abstract

Short-term exposure to traffic-related gaseous pollutants [nitrogen dioxide (NO_2_) and sulfur dioxide (SO_2_)] may increase childhood bronchopneumonia risk, but evidence from megacities with high pollution levels remains limited. This study aimed to evaluate the association between short-term exposure to NO_2_ and SO_2_ and pediatric bronchopneumonia hospitalization in Guangzhou. We collected all pediatric inpatient records with a primary discharge diagnosis of bronchopneumonia (International Classification of Diseases, Tenth Revision: J18) among children aged 0 to 14 years from a tertiary hospital in the Huadu District, between 1 January 2014 and 31 December 2023. Daily air pollutant and meteorological data were integrated with admission records and analyzed using a generalized additive model with quasi-Poisson distribution. A total of 20,623 hospitalisations were included, of which 60.6% were male and 68.4% were aged <6 years. Both NO_2_ and SO_2_ were positively associated with bronchopneumonia hospitalization. Per 5 μg/m^3^ increase in pollutant concentration, for single-day exposure, the peak excess risks (ERs) occurred at lag 4 (NO_2_: 2.74% [95% confidence interval (CI): 1.73–3.75]; SO_2_: 8.81% [95% CI: 4.94–12.82]); For the 0-7 day moving average, the ERs increased to 6.83% (95% CI 4.93–8.77) for NO_2_ and 17.21% (95% CI 10.18–24.70) for SO_2_. Children aged 6–14 years and boys showed consistently higher susceptibility. These findings provide the first long-term evidence from Guangzhou that short-term NO_2_ and SO_2_ exposure is positively associated with childhood bronchopneumonia hospitalization, with older children and boys being the susceptible subgroups. Public health authorities should consider developing targeted air pollution alert thresholds and intervention strategies for school-aged children and boys.

## 1 Introduction

Childhood bronchopneumonia, the most common clinical phenotype of community-acquired pneumonia, remains a major contributor to global pediatric morbidity and mortality. According to the Global Burden of Disease Study 2023, lower respiratory infections caused 2.5 million deaths worldwide, with children aged under 5 years carrying a disproportionate burden at a mortality rate of 94.8 per 100,000 person-years (1). In high-income settings, although mortality has declined, pneumonia still accounts for substantial hospitalisations—approximately 150 000 annual hospitalisations among United States children (2). The challenge is even greater in low- and middle-income countries. Historical estimates indicate that China ranked second globally for pneumococcal disease burden in children aged under 5 years, accounting for about 12% of worldwide cases (3). However, contemporary national incidence data remain scarce. Therefore, updated clinical and epidemiological characterization of childhood bronchopneumonia is urgently needed to inform targeted prevention and treatment strategies.

Epidemiological studies on ambient air pollution and hospitalisations for childhood bronchopneumonia or related respiratory tract infections have increased substantially, yet the findings remain notably inconsistent, particularly with regard to traffic-related gaseous pollutants such as nitrogen dioxide (NO_2_) and sulfur dioxide (SO_2_). Effect estimates vary considerably across countries and regions: a European birth cohort meta-analysis reported an adjusted odds ratio of 1.30 [95% confidence interval (CI): 1.02–1.65] for pneumonia per 10 μg/m^3^ increase in NO_2_ (4), whereas a time-series study in Delhi, India, found a 13.15% increase in pediatric emergency visits per 10 μg/m^3^ increase in SO_2_ (5). Substantial heterogeneity also exists within China: a nine-city study in Southwest China showed relative risks of 1.0278 and 1.0378 per 1 μg/m^3^ increase (6); and a meta-analysis of nearly 900,000 hospitalisations across 25 major Chinese cities found that each 10 μg/m^3^ increase in NO_2_ and SO_2_ was associated with a 0.54% and 0.60% rise in all-cause respiratory infection hospitalisations, respectively (7). Furthermore, subgroup findings are inconsistent: some studies report higher vulnerability in younger children (particularly those under 5 years of age) (8), while others suggest that girls may be at greater risk (9). These inconsistencies highlight the need for additional high-quality time-series studies to further clarify the true association between ambient air pollution exposure and hospitalization for childhood bronchopneumonia.

However, as a megacity in the Pearl River Delta with prominent traffic-related pollution (10) and a national hotspot for both NO_2_ and SO_2_ co-pollution confirmed by remote sensing (11), Guangzhou still lacks long-term time-series evidence specifically targeting the risk of childhood bronchopneumonia hospitalization. Moreover, existing findings from other Chinese cities show considerable heterogeneity in effect sizes and subgroup susceptibility, making the evidence gap in Guangzhou particularly salient. To address this gap, the present study leveraged 20,623 pediatric bronchopneumonia hospitalisations in Guangzhou from 2014 to 2023 and employed a generalized additive model to systematically evaluate the association between short-term exposure to NO_2_ and SO_2_ and hospitalization risk. By covering a 10-year observation period and focusing on daily exposure and lag effects, this study aims to provide robust local epidemiological evidence for region-specific air quality management and precision prevention of childhood respiratory infections——for instance, establishing evidence-based air pollution alert thresholds and seasonal intervention strategies.

## 2 Materials and methods

### 2.1 Data collection

This study employed a time-series design. We collected all pediatric inpatient records with a primary discharge diagnosis of bronchopneumonia [International Classification of Diseases, Tenth Revision (ICD-10): J18] among children aged 0 to 14 years from the Huadu District Maternal and Child Healthcare Hospital in Guangzhou, between 1 January 2014 and 31 December 2023. Data were extracted from the electronic medical record system of this institution. All study subjects were residents of Guangzhou City. This hospital is a tertiary-level maternal and child healthcare hospital in the Huadu District of Guangzhou, serving as a regional pediatric care center, with its primary service population comprising permanent residents of Huadu District and its surrounding urban areas. Daily numbers of hospitalized bronchopneumonia cases were calculated and then divided into two strata by sex (male, female) and age group: younger (<6 years) and older (≥6 years). The study protocol was reviewed and approved by the Ethics Committee of The Maternal and Children Health Care Hospital (Huzhong Hospital) of Huadu, Guangzhou, Guangdong, China (approval No. 2026-030). Because this study involved secondary use of de-identified, aggregated electronic medical records, the requirement for written informed consent was waived by the committee.

Daily concentrations of NO_2_, SO_2_, fine particulate matter (PM_2.5_, atmospheric particulates with aerodynamic diameter smaller than 2.5 μm), inhalable particulate matter (PM_10_, atmospheric particulates with aerodynamic diameter smaller than 10 μm), and ozone (O_3_) in Guangzhou were obtained from the ChinaHighAirPollutants (CHAP) dataset (12–16), a high-resolution gridded dataset generated from multi-source data using artificial intelligence. For each pollutant, we extracted daily estimates from all grid cells within Guangzhou's administrative boundaries and calculated the city-wide daily mean as the population-level exposure metric. This city-wide average was chosen because time-series designs primarily exploit temporal rather than spatial variations in exposure. Independent validation has demonstrated high accuracy, with cross-validated R^2^ values ranging from 0.84 to 0.93. To control for potential confounding effects of meteorological variables, daily meteorological reanalysis data for Guangzhou—including mean 2 m temperature and mean 2 m relative humidity-were obtained from the National Aeronautics and Space Administration Prediction of Worldwide Energy Resources website, and incorporated into the analysis to adjust for these meteorological factors. Prior to analysis, we systematically verified the completeness of all datasets and confirmed that no daily observations were missing for any pollutant or meteorological variable across the entire 10-year study period.

### 2.2 Statistical analyses

This study used a time-series design to evaluate the association between short-term exposure to ambient air pollutants (NO_2_ and SO_2_) and childhood bronchopneumonia. First, descriptive analyses were performed to calculate the mean, standard deviation, quantiles, and other summary statistics for daily hospital admissions for childhood bronchopneumonia, daily mean concentrations of each air pollutant, and daily meteorological variables. Spearman's rank correlation was used to examine correlations among variables, and variables with correlation > 0.8 were excluded to avoid multicollinearity. An overdispersion test confirmed that the daily admission counts exhibited significant overdispersion (dispersion = 1.246, p < 0.001), and exploratory analyses suggested non-linear relationships with covariates. Accordingly, a generalized additive model (GAM) with a quasi-Poisson distribution was applied, as this framework simultaneously corrects for overdispersion and flexibly adjusts for non-linear confounding through natural cubic splines. The GAM formula is as follows:


$$\begin{array}{l} ln\left[E\left(Yt\right)\right]\;=\;\alpha \;+\;\beta Z_{t}\;+\;DOW_{t}\;+\;ns\;\left(time,\;df\right)\;\\ +ns\;\left(temperature,\;df\right)\;+\;ns\;\left(relative\;humidity,\;df\right) \end{array}$$

where *E*(*Y*_*t*_) denotes the expected number of hospital admissions for bronchopneumonia on day *t* (*t* = 1, 2, …, *T*, \with *T*= 3,652 corresponding to the total number of days from 1 January 2014 to 31 December 2023); α is the intercept; Z_*t*_ represents the exposure concentration of NO_2_ or SO_2_ on day *t*; β is the log linear coefficient representing the change in the log expected number of admissions per unit increase in *Z*_*t*_; DOW_*t*_ is a factor variable for day of the week, used to adjust for within-week effects; *ns* (*time, df* ) is a natural cubic spline smoothing function of date (where *df* denotes degrees of freedom), employed to control for long-term trends and seasonality, and by referring to existing research literature, 7 *df* per year were selected for the time variable; and *ns* (*temperature, df* ) and *ns* (*relative humidity, df* ) are natural cubic spline smoothing functions for daily mean temperature and daily mean relative humidity, respectively, with 3 *df* selected for both (17, 18).

To investigate the lagged effects of air pollution, single-day lag variables (lag0 to lag7; lag0 = concentration on the current day, lag7 = concentration seven days prior) and multi-day moving average lag variables (lag01 to lag07; lag01 = two-day rolling mean of current and previous day, lag07 = eight-day rolling mean of current and previous seven days) were constructed for NO_2_ and SO_2_. These were analyzed in single-pollutant models with bronchopneumonia as the outcome. Based on the results of the single-pollutant models, for each pollutant we selected the lag day with the largest absolute excess risk (ER) that was also statistically significant as the optimal lag variable. This was used to estimate the association between NO_2_ and SO_2_ and the risk of hospitalization for bronchopneumonia. Results are expressed as the percentage ER and 95% CI per 5 μg/m^3^ increase in pollutant concentration. The excess risk percentage was derived from the GAM regression coefficient β using the formula:


$$\begin{array}{l} ER\;=\left(e^{\beta \times △}-1\right)\;\times 100\% \end{array}$$

where Δ = 5 μg/m^3^. The 95% CI was calculated as:


$$\begin{array}{l} \left(e^{\beta \pm 1.96\times SE}-1\right)\;\times 100\% \end{array}$$

where SE is the standard error of β.

To examine the exposure-response relationship, natural cubic splines with 3 df were applied to NO_2_ and SO_2_ concentrations (lag0) within the same GAM framework, with the 2.5th percentile concentration as the reference. The log relative risks and 95%CI were then plotted. Prior to sensitivity analyses, we performed model diagnostics—including autocorrelation function (ACF) plots, residual plots, and quantile-quantile (Q-Q) plots, overdispersion parameters, and checks for residual-covariate associations—all of which confirmed an adequate quasi-Poisson fit and supported the validity of our confounding adjustment. To assess the robustness of the findings, a series of sensitivity analyses were conducted. First, two-pollutant models were fitted for three fixed lag variables (lag1, lag02, and lag07) to examine the associations of NO_2_ and SO_2_ with bronchopneumonia after adjusting for PM_2.5_, PM_10_, or O_3_ individually. Second, stratified analyses were performed by sex and age subgroups. Third, to minimize the potential impact of the COVID-19 pandemic, we repeated the main analyses using the same three fixed lag structures (lag1, lag02, and lag07) after excluding data from 2020 to 2022; the effect estimates remained comparable to those derived from the full dataset. All statistical analyses were conducted using R software (version 4.5.0) and its core packages, including mgcv and dplyr.

## 3 Results

Between January 2014 and December 2023, a total of 20,623 childhood bronchopneumonia cases were identified, with a consistent male predominance (approximately 3:2 ratio) throughout the study period. Temporally, the annual caseload exhibited marked fluctuations, plummeting to a nadir of 999 in 2020 amidst the COVID-19 pandemic restrictions, before rebounding sharply to a peak of 3,021 in 2023. Notably, contrary to the relatively stable sex distribution, the age composition underwent a striking reversal over time: while children aged < 6 years overwhelmingly dominated the early years (peaking at >78% in 2018), their proportion steadily declined to 44.8% in 2023, concurrently allowing the 6–14 years group to surpass them and become the majority (55.2%) for the first time, suggesting a post-pandemic epidemiological shift in pathogen exposure among school-aged children (Table 1).

**Table 1:** Annual number and proportion of childhood bronchopneumonia cases by sex and age group, 2014–2023.

Group	2014	2015	2016	2017	2018	2019	2020	2021	2022	2023	Total
Total	1,811	1643	2069	1878	3371	3194	999	1321	1316	3021	20623
**Sex**											
Male	1,096 (60.5%)	979 (59.6%)	1,245 (60.2%)	1,102 (58.7%)	2,109 (62.6%)	2,015 (63.1%)	602 (60.3%)	819 (62.0%)	775 (58.9%)	1,756 (58.1%)	12,498 (60.6%)
Female	715 (39.5%)	664 (40.4%)	824 (39.8%)	776 (41.3%)	1,262 (37.4%)	1,179 (36.9%)	397 (39.7%)	502 (38.0%)	541 (41.1%)	1,265 (41.9%)	8,125 (39.4%)
**Age**											
6–14 years	576 (31.8%)	512 (31.2%)	580 (28.0%)	420 (22.4%)	714 (21.2%)	785 (24.6%)	262 (26.2%)	472 (35.7%)	537 (40.8%)	1,668 (55.2%)	6,526 (31.6%)
< 6 years	1,235 (68.2%)	1,131 (68.8%)	1,489 (72.0%)	1,458 (77.6%)	2,657 (78.8%)	2,409 (75.4%)	737 (73.8%)	849 (64.3%)	779 (59.2%)	1,353 (44.8%)	14,097 (68.4%)

Data are presented as *n* (%), where the percentage for each year represents the proportion within that year's total cases.

**Figure 1:**
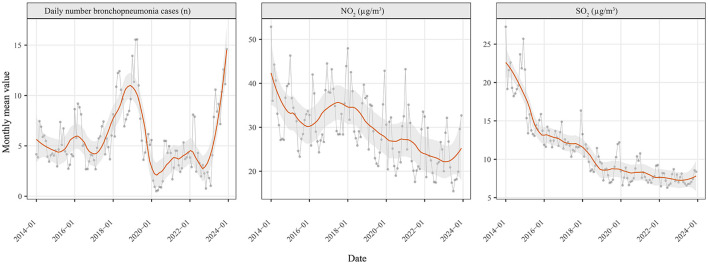
Monthly aggregated time-series trends of daily bronchopneumonia cases, ambient air pollutants, and meteorological factors from 2014 to 2023. Gray dots and thin lines represent monthly means, the orange solid curve indicates the locally weighted regression-smoothed trend, and the gray shaded ribbon denotes the 95% confidence interval. Each panel has an independent y-axis scale, and the *x*-axis indicates the year of monthly aggregated data. *NO*_2_, nitrogen dioxide; *SO*_2_, sulfur dioxide.

From 2014 to 2023, the daily number of bronchopneumonia cases in Guangzhou exhibited a winter peak similar to those of NO_2_ and SO_2_; however, the long-term trends of these two pollutants diverged markedly. SO_2_ declined from approximately 27 μg/m^3^ to 8 μg/m^3^, whereas NO_2_ decreased only modestly, from 53 μg/m^3^ to 33 μg/m^3^. Over the same period, PM_2.5_ and PM_10_ also showed sustained reductions, falling from about 80 and 110 μg/m^3^ to < 30 and < 50 μg/m^3^, respectively. O_3_ displayed a seasonal pattern opposite to that of the combustion-related pollutants, with no obvious long-term trend. Temperature and humidity were dominated by seasonal fluctuations with little interannual variation. Case numbers were markedly reduced from February to August 2020, possibly related to COVID-19 control measures, and then rebounded sharply from September to December 2023, suggesting a potential post-pandemic resurgence. The concurrent wintertime elevation of both cases and combustion pollutants implies a possible seasonal synergistic effect, warranting further investigation (Figure 1, Supplementary Figure S1, and Table 2).

**Table 2:** Descriptive statistics of daily bronchopneumonia hospitalisations, ambient air pollutant concentrations, and meteorological variables during the study period (2014–2023).

Variable	Mean	SD	Min	P25	Median	P75	Max
Bronchopneumonia cases (*n*/day)	5.65	4.17	0.00	3.00	5.00	8.00	26.00
NO_2_ (μg/m^3^**)**	29.80	10.99	6.69	21.96	27.85	35.35	89.71
SO_2_ (μg/m^3^**)**	11.13	5.01	5.28	7.44	9.58	13.17	40.21
PM_2.5_ (μg/m^3^**)**	29.66	16.18	2.79	17.75	26.31	38.06	133.01
PM_10_ (μg/m^3^**)**	48.27	22.21	5.13	31.91	44.09	61.03	162.61
O_3_ (μg/m^3^**)**	98.20	40.80	14.72	66.59	95.48	125.38	252.08
Temp (°C)	23.06	5.78	2.90	19.00	24.70	28.00	32.10
RH (%)	78.46	10.67	24.30	72.50	80.70	86.50	95.30

SD, standard deviation; Min, minimum; P25, 25th percentile; P75, 75th percentile; Max, maximum; NO_2_, nitrogen dioxide; SO_2_, sulfur dioxide; PM_2.5_, fine particulate matter with aerodynamic diameter ≤ 2.5 μm; PM_10_, particulate matter with aerodynamic diameter ≤ 10 μm; O_3_, ozone; Temp, temperature; RH, relative humidity.

As presented in Supplementary Table S1, NO_2_ and SO_2_ were strongly positively associated with PM_2.5_ and PM_10_ (r = 0.720–0.866), whereas their correlations with O_3_ were weaker, and they showed weak-to-moderate negative correlations with temperature and relative humidity. In particular, PM_2.5_ and PM_10_ exhibited an extremely strong positive correlation (r = 0.976). Owing to the substantial collinearity between these two variables, they were not entered simultaneously into any regression model.

**Figure 2:**
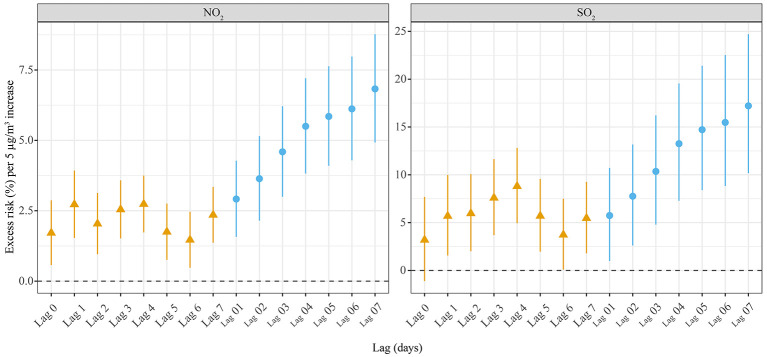
Lag-specific and cumulative excess risks of childhood bronchopneumonia hospitalisations associated with a 5 μg/m^3^ increase in NO_2_ and SO_2_. Points represent the percent excess risk; vertical lines indicate 95% confidence intervals. The horizontal dashed line marks zero excess risk.

Positive associations were observed for NO_2_ and SO_2_ with bronchopneumonia hospitalization risk (Figure 2, Supplementary Table S2). For single-day lags, tboth NO_2_ and SO_2_ exhibited the strongest effects at lag day 4, with excess risks per 5 μg/m^3^ increase of 2.74% (95% CI: 1.73–3.75) for NO_2_ and 8.81% (95% CI: 4.9–412.82) for SO_2_. The effect estimates for cumulative exposure were substantially larger and increased progressively with longer averaging periods, peaking at the 0-7 day moving average, where the excess risks per 5 μg/m^3^ increase were 6.83% (95% CI: 4.93–8.77) for NO_2_ and 17.21% (95% CI: 10.18–24.70) for SO_2_. Overall, the effect estimates for SO_2_ were consistently higher than those for NO_2_, suggesting that sulfur-containing pollutants have a more pronounced impact on the respiratory system.

**Figure 3:**
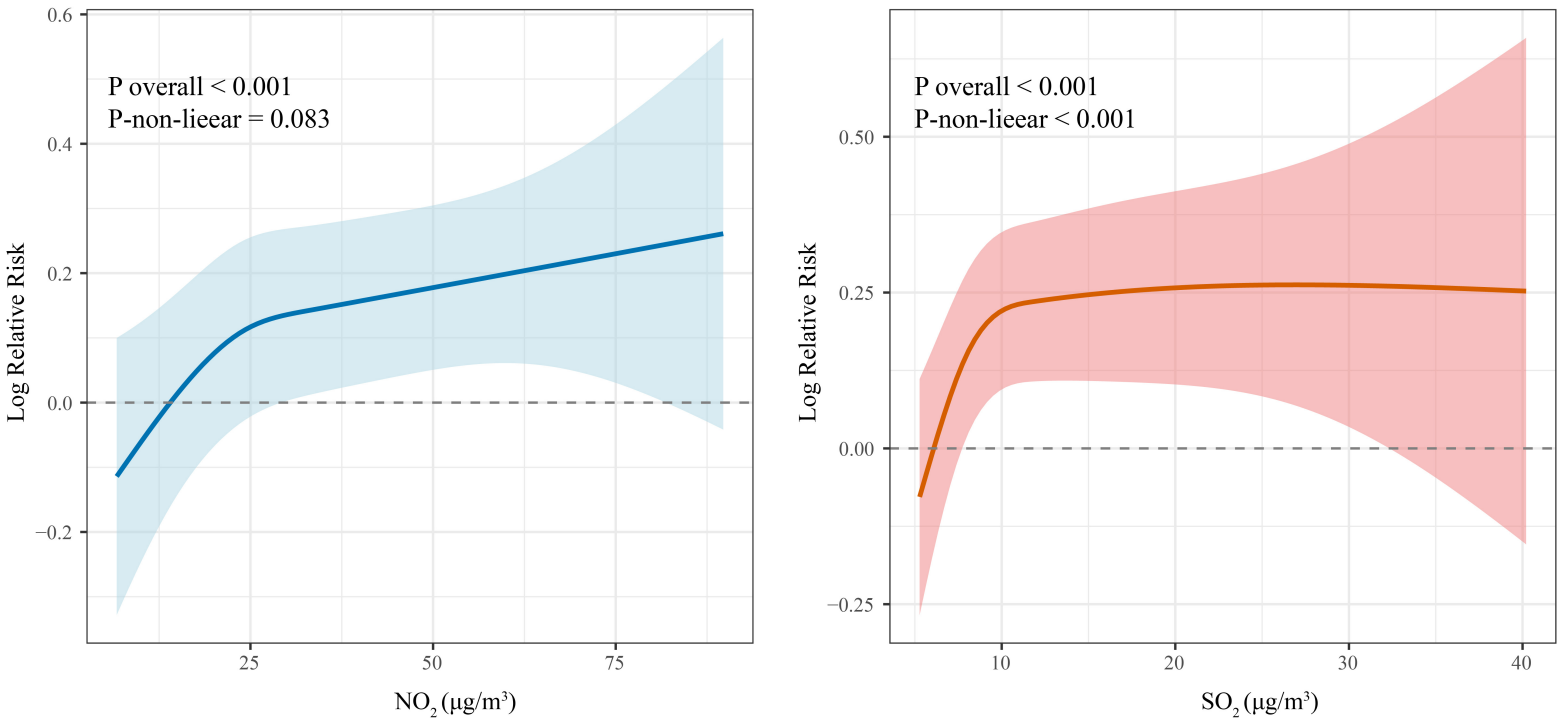
Exposure–response curves for NO_2_ and SO_2_ and childhood bronchopneumonia hospitalizations. Solid lines represent the estimated log relative risk; shaded bands indicate 95% confidence intervals. The reference concentration for each pollutant was set at the 2.5th percentile of its distribution.

As shown in Figure 3, SO_2_ exhibited a significant non-linear exposure–response relationship (*P* < 0.001), with the curve rising steeply at low concentrations before leveling off, suggesting a saturation effect whereby the majority of the excess risk accumulates within relatively low exposure ranges. In contrast, NO_2_ showed an approximately linear trend (*P* = 0.083); however, the overall associations for both pollutants were statistically significant (*P* < 0.001). Notably, the non-linear pattern for SO_2_ implies that reducing concentrations from moderate to low levels may yield greater risk reductions than further decreases at the lower tail, highlighting a potentially efficient intervention window.

Diagnostic checks confirmed adequate model fit: residuals were approximately independent and homoscedastic, with no significant autocorrelation or systematic association with any predictor. The dispersion parameters were 1.32 for both models, supporting the quasi-Poisson specification (Supplementary Figures S2, S3). In two-pollutant models, the positive associations of NO_2_ with bronchopneumonia hospitalisations remained significant after adjusting for PM_2.5_, PM_10_, or O_3_, with effect estimates increasing progressively across averaging periods and maximizing at lag07 (largest: 7.88% (95% CI: 5.36–10.46) after adjusting for PM_2.5_). By contrast, for SO_2_, statistical significance was attenuated after adjusting for co-pollutants, with no significant effects at lag1 or lag02; only at lag07 did the association remain significant (largest: 16.80% (95% CI: 6.87–27.65) after adjusting for PM_2.5_). This contrast suggests that the SO_2_ effect may be partially confounded or mediated by particulate matter, whereas the NO_2_ association appears more robust to co-pollutant adjustment (Table 3).

**Table 3:** Excess risks (ER%) and 95% confidence intervals for childhood bronchopneumonia hospitalization associated with a 5 μg/m^3^ increase in NO_2_ and SO_2_ in two-pollutant models at different lag days.

Pollutant	ER% (95% CI)		
	Lag 1	Lag02	Lag07
NO_2_+PM_2.5_	2.31 (0.81, 3.83)	3.29 (1.40, 5.22)	7.88 (5.36, 10.46)
NO_2_+PM_10_	2.25 (0.67, 3.85)	3.09 (1.07, 5.16)	7.05 (4.40, 9.76)
NO_2_+O_3_	2.28 (1.05, 3.53)	2.97 (1.38, 4.58)	6.04 (4.02, 8.09)
SO_2_+PM_2.5_	1.51 (−3.79, 7.11)	3.05 (−3.63, 10.19)	16.80 (6.87, 27.65)
SO_2_+PM_10_	0.53 (−5.02, 6.40)	1.10 (−5.82, 8.52)	10.82 (0.75, 21.88)
SO_2_+O_3_	2.71 (−1.79, 7.43)	3.72 (−1.94, 9.70)	12.13 (4.03, 20.87)

Lag1, single-day lag (1 day prior); Lag02, 0–2 day moving average; Lag07, 0–7 day moving average. ER, excess risk; 95%CI, 95%confidence intervals. NO_2_, nitrogen dioxide; SO_2_, sulfur dioxide; PM_2.5_, fine particulate matter with aerodynamic diameter ≤ 2.5 μm; PM_10_, particulate matter with aerodynamic diameter ≤ 10 μm; O_3_, ozone.

**Figure 4:**
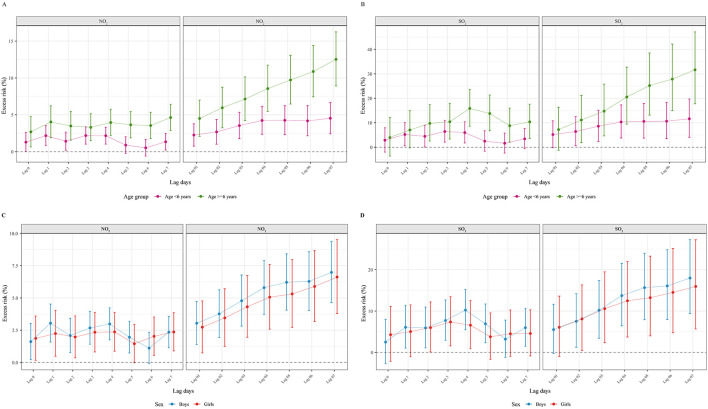
Age- and sex-stratified excess risks (ER, %) of bronchopneumonia hospitalisations per 5 μg/m^3^ increase in NO_2_ and SO_2_ across single-day lags and cumulative moving average lags. **(A, B)** Age-stratified results for NO_2_ and SO_2_, respectively. **(C, D)** Sex-stratified results for NO_2_ and SO_2_, respectively. Points represent point estimates; error bars indicate 95% confidence intervals; the horizontal dashed line marks zero ER.

Subgroup analyses revealed that the association between gaseous pollutants and bronchopneumonia hospitalization was consistently stronger in children aged 6–14 years than in those under 6 years across both single-day and cumulative lag models. For every 5 μg/m^3^ increase in NO_2_ or SO_2_, the ER in the older children was approximately double that in the younger children. The most pronounced risks emerged from the 7-day moving average exposure (Lag 07), with the 6–14 years group showing ERs of 12.53% (95% CI: 8.92–16.25) for NO_2_ and 31.66% (95% CI: 17.78–47.17) for SO_2_, substantially exceeding the corresponding risks of 4.53% (95% CI: 2.43, 6.68) and 11.63% (95% CI: 4.08, 19.71) in the < 6 years group. Sex-stratified analyses revealed a parallel pattern, with boys exhibiting generally higher susceptibility than girls, particularly across cumulative lag structures (Figure 4, Supplementary Tables S3–S4).

As presented in Supplementary Table S5, excluding 2020–2022 attenuated the ERs for both pollutants, but the overall pattern and significance persisted for NO_2_ (Lag1, Lag02, Lag07) and SO_2_ (Lag1, Lag07), except for SO_2_-Lag02 which became borderline non-significant. These results confirm the main findings are robust to pandemic-period exclusion

## 4 Discussion

To the best of our knowledge, this is the first time-series study conducted in Guangzhou to simultaneously assess the association between short-term exposure to traffic-related pollutants, specifically NO_2_ and SO_2_, and the risk of hospitalization for childhood bronchopneumonia, with a particular focus on multi-day cumulative lag effects and exposure-response relationships. The study included 20,623 children hospitalized for bronchopneumonia between 2014 and 2023, representing one of the largest pediatric samples for this condition in southern China over the past decade. The main findings indicate that short-term exposure to these two traffic-related pollutants is significantly positively associated with the risk of hospitalization for childhood bronchopneumonia, and that the effect sizes of cumulative exposure are greater than those of single-day exposure. Stratified analyses suggest that children aged 6 years and older, as well as boys, are more susceptible to the health effects of traffic-related pollutants.

Our findings are consistent with multiple domestic and international time-series studies. A time-series study from Lanzhou, China, found that short-term exposure to SO_2_ and NO_2_ was significantly positively associated with the risk of hospitalization for acute lower respiratory tract infection in children aged 0–14 years, with relative risks at lag 0–7 days of 1.186 and 1.149, respectively (19). In a multicentre time-series study covering 192,079 hospitalisations for childhood acute lower respiratory tract infection in Southwest China, each 10 μg/m^3^ increase in pollutant concentration corresponded to a relative risk of pneumonia hospitalization of 1.0278 for SO_2_ (per 1 μg/m^3^) and 1.0378 for NO_2_, and children aged 5–14 years were more susceptible than those aged 0–4 years (6). A meta-analysis based on nearly 900,000 hospitalisations for childhood respiratory tract infection in 25 major Chinese cities reported that each 10 μg/m^3^ increase in NO_2_ and SO_2_ (lag 01 day) was associated with a 0.54% and 0.60% increase in the risk of all-cause respiratory tract infection, respectively (7). Notably, the effect estimates for SO_2_ and NO_2_ in our study are higher than those reported in most earlier studies. Several factors may explain this difference. First, air pollution profiles vary across cities: Guangzhou is located in the economically developed Pearl River Delta region, where industrial emissions and vehicular exhaust coexist, and the source composition may differ from that in central or western Chinese cities. Second, differences in the study period may influence effect estimation. Our study covers 10 years of data from 2014 to 2023, with a sample size of 20,623 children, and the changes in exposure patterns over such a long period may affect the magnitude of effect estimates. Furthermore, our study used a specific outcome—childhood bronchopneumonia (ICD-10: J18.0)-whereas some previous studies used broader outcomes such as acute lower respiratory tract infection or all-cause respiratory tract infection; such differences in outcome definition may also contribute to heterogeneity in effect estimates. It should be added that although positive associations between air pollution and childhood respiratory diseases in Guangzhou have been reported in previous studies (20–22), their outcomes were respiratory pathogen infection, broad respiratory diseases, and non-specific pneumonia, respectively, and none systematically evaluated the independent effect of SO_2_. In contrast, the present study is the first to focus specifically on the long-term time-series association between NO_2_ and SO_2_ and the risk of hospitalization for childhood bronchopneumonia, thereby filling an evidence gap for this specific phenotype.

The present study found that children aged 6–14 years exhibited significantly higher susceptibility to NO_2_ and SO_2_ than those aged < 6 years, which is consistent with the findings from a previous multicentre study showing greater susceptibility to PM_2.5_, SO_2_ and NO_2_ in children aged 4–14 years (23), as well as the results of a study in Jinan showing that schoolchildren were more sensitive to PM_2.5_ than infants and toddlers (24). Furthermore, our results showed higher effect estimates for NO_2_ and SO_2_ in male children, aligning with the findings from the Jinan study, which reported a 0.32% increase in hospitalization risk in males with a 95% confidence interval of 0.04%−0.06% (24), and a study in Wuhan demonstrating that NO_2_ and SO_2_ were significantly associated with pneumonia hospitalisations only in males (25). Collectively, these findings indicate age- and sex-related differences in the susceptibility of the pediatric respiratory system to air pollution, which may be attributed to multiple factors. Older children have a higher lung ventilation per unit body weight and spend more time outdoors, leading to increased exposure dose; additionally, the lungs and immune system remain actively developing during the school-age years. Moreover, male children may receive a higher inhaled dose due to their larger body surface area and higher minute ventilation, while sex chromosomes may mediate sex-specific lung injury and repair responses – for example, glutathione S-transferase P1 hypermethylation induced by PM_2.5_ exposure during late pregnancy has been observed predominantly in male offspring (26). These behavioral and biological mechanisms collectively contribute, at least in part, to the greater susceptibility observed in older and male children.

Although the causal relationship between short-term exposure to NO_2_ and SO_2_ and the risk of hospitalization for bronchopneumonia in children has not yet been fully elucidated, existing studies have provided several important clues regarding the underlying biological pathways, including oxidative stress, airway inflammatory responses, and immune dysfunction (27, 28). Upon entering the respiratory tract, air pollutants can induce excessive generation of reactive oxygen and nitrogen species; when pulmonary antioxidant capacity is exceeded, an oxidative stress state ensues, leading to lipid peroxidation, cellular damage, and impaired mucociliary clearance. Animal experiments have confirmed that exposure to oxidative air pollutants can induce pulmonary redox imbalance and dysregulation of the renin–angiotensin–aldosterone system, thereby compromising innate immune function (29). Furthermore, epidemiological evidence indicates a significant positive correlation between NO_2_ exposure and the risk of pneumococcal pneumonia in children, as well as an increase in the minimum inhibitory concentration of penicillin against *Streptococcus pneumoniae* (30), suggesting that NO_2_ may indirectly exacerbate infection severity by enhancing pathogen invasiveness. In addition, NO_2_ exposure can impair the lung's ability to clear pathogens by interfering with innate immune signaling, upregulating the inflammatory microenvironment, and suppressing regulatory T-cell responses. Moreover, NO_2_ exposure upregulates the expression of intercellular adhesion molecule-1 on airway epithelial cells—a key receptor for several respiratory viruses, such as rhinovirus and respiratory syncytial virus—thereby increasing susceptibility to viral infection. These mechanisms have been substantiated in controlled human exposure studies involving healthy bronchial epithelium (31). Given that the lungs and immune system in children are still developing, their capacity to eliminate oxidative damage is relatively weak, and their metabolic rate and respiratory frequency per unit body-to-body-weight ratio are higher than those in adults, rendering them particularly vulnerable to gaseous pollutant exposure (28). This cascade of reactions may collectively explain the association between short-term air pollution exposure and an elevated risk of hospitalization for childhood bronchopneumonia. However, the aforementioned mechanisms are primarily based on evidence from adult populations and animal experiments; whether NO_2_ and SO_2_ specifically affect children's susceptibility to respiratory infections through pathways such as airway microbiota remodeling or epigenetic regulation remains to be further elucidated.

The sensitivity analyses confirmed the robustness of the main findings. In two-pollutant models, NO_2_ remained significant, whereas SO_2_ was significant only at lag07, highlighting that cumulative exposure is key to detecting its independent effect. This differential pattern may reflect the distinct physicochemical properties and deposition characteristics of the two pollutants in pediatric airways. Moreover, the associations were largely unchanged after excluding 2020–2022, with only marginal SO_2_ attenuation at lag02, suggesting that pandemic-related disruptions did not drive the main results. Stratified analyses confirmed higher susceptibility in older children and boys, pointing to the need for targeted prevention. Overall, these findings reinforce that multi-day NO_2_ and SO_2_ exposure independently increases hospitalization risk for childhood bronchopneumonia, with SO_2_ effects being more dependent on cumulative exposure.

Although this study employed a rigorous time-series design and robust statistical methods to reveal a positive association between short-term exposure to NO_2_ and SO_2_ and the risk of hospitalization for bronchopneumonia in children, the findings should be interpreted with caution given the following limitations. First, there is an inherent ecological fallacy in time-series studies of air pollution. This study used the city-wide daily mean concentrations from the CHAP dataset as a surrogate for population-level exposure, which may not reflect the true exposure dose at the individual level. Moreover, actual exposure experienced by individuals may differ from monitored values due to variations in daily activity patterns; the resulting exposure misclassification could lead to biased effect estimates (32). Nevertheless, this exposure misclassification is likely non-differential with respect to hospitalization outcomes, which would typically bias effect estimates toward the null; therefore, our significant findings likely represent conservative estimates of the true associations. Second, although meteorological factors such as temperature, relative humidity and wind speed were included, other potential confounders could not be ruled out, including indoor air pollution, household cooking and heating practices, tobacco exposure, socioeconomic status, nutritional status, and vaccination history. These variables may exert confounding effects on the risk of hospitalization for respiratory infections (33–35). Third, we acknowledge that the study data were derived solely from Guangzhou, and hospitalization cases were limited to this single institution, which did not encompass all children hospitalized for bronchopneumonia in the city, thereby restricting the generalisability of our findings to primary care settings, rural communities, or regions with markedly different climatic conditions (e.g., northern Chinese cities with cold, dry winters) or alternative healthcare access models. Nevertheless, Huadu District accounts for approximately 9.3% of Guangzhou's permanent population, and this suburban population has been largely underrepresented in previous studies that relied on central urban monitoring stations; our findings thus provide the first long-term evidence for this demographic, filling an important geographical gap in the existing literature. In addition, this study only included children hospitalized for bronchopneumonia (ICD-10: J18), excluding mild cases treated in outpatient clinics or those managed at home without medical consultation, which may underestimate the true disease burden attributable to air pollution. Overall, while these limitations are unavoidable, the findings of this study provide important epidemiological evidence for understanding the acute effects of air pollution on hospitalization for bronchopneumonia in children. Future validation of these results in large-scale, multicentre studies across diverse climate zones and healthcare tiers is warranted.

## 5 Conclusion

This study provides the first long-term time-series evidence from Guangzhou on the acute effects of traffic-related gaseous pollutants on childhood bronchopneumonia hospitalization. The findings underscore that c measures, though beneficial, may not fully protect pediatric respiratory health against the acute impacts of NO_2_ and SO_2_. Public health authorities should consider developing more nuanced air pollution alert thresholds and implementing season-specific intervention strategies targeting high-risk groups, such as school-aged children and boys. Future research incorporating individual-level exposure assessment and additional confounders (e.g., indoor air quality, socioeconomic status) is needed to further validate and refine these estimates.

## Data Availability

The raw data supporting the conclusions of this article will be made available by the authors, without undue reservation.
