# Supplementary material for: Short-term traffic-related gaseous pollutant exposure is associated with childhood bronchopneumonia hospitalization: a 10-year time-series study from Guangzhou, China
Source: Front Public Health. 2026 Jul 16;14:1885590. doi: 10.3389/fpubh.2026.1885590 (PMC13422424; doi:10.3389/fpubh.2026.1885590)
Supplement: Supplementary file 1 [file Data_Sheet_1.docx]

**Supplementary material**

[Table S1. Spearman correlation matrix for ambient air pollutants and meteorological variables 1](#_Toc233193024)

[Table S2. Eexcess risks (ER%) and 95% confidence intervals (95% CI) for childhood bronchopneumonia hospitalization associated with a 5 μg/m^3^ increase in NO_2_ and SO_2_ at different lag days and moving averages. 2](#_Toc233193025)

[Table S3. Age‑stratified excess risks (ER%) and 95% confidence intervals (95% CI) for childhood bronchopneumonia hospitalization associated with a 5 μg/m^3^ increase in NO_2_ and SO_2_ 4](#_Toc233193026)

[Table S4. Sex‑stratified excess risks (ER%) and 95% CI for childhood bronchopneumonia hospitalization associated with a 5 μg/m³ increase in NO_2_ and SO_2_ 5](#_Toc233193027)

[Table S5. Excess Risks (ER) and 95% Confidence Intervals (CI) for NO_2_ and SO_2_ exposure across different lag structures, stratified by study period. 6](#_Toc233193028)

[Figure S1. Annual January trends of air pollutant concentrations and meteorological parameters from 2014 to 2023. 7](#_Toc233193029)

[Figure S2. Autocorrelation function (ACF) plots of deviance residuals from the single-pollutant GAM models. 8](#_Toc233193030)

[Figure S3. Residual diagnostic plots for the single-pollutant GAM models. 9](#_Toc233193031)

# Table S1. Spearman correlation matrix for ambient air pollutants and meteorological variables

|  | NO_2_ | SO_2_ | PM_2.5_ | PM_10_ | O_3_ | Temp | RH |
| --- | --- | --- | --- | --- | --- | --- | --- |
| NO_2_ | 1.000 |  |  |  |  |  |  |
| SO_2_ | 0.720^*^ | 1.000 |  |  |  |  |  |
| PM_2.5_ | 0.845^*^ | 0.816^*^ | 1.000 |  |  |  |  |
| PM_10_ | 0.866^*^ | 0.800^*^ | 0.976^*^ | 1.000 |  |  |  |
| O3 | -0.410^*^ | -0.215^*^ | -0.264^*^ | -0.177 | 1.000 |  |  |
| Temp | -0.615^*^ | -0.284^*^ | -0.631^*^ | -0.594^*^ | 0.556^*^ | 1.000 |  |
| RH | -0.470^*^ | -0.135 | -0.531^*^ | -0.572^*^ | 0.069 | 0.714^*^ | 1.000 |

^*^: P < 0.05

**Abbreviations:** *NO_2_*, nitrogen dioxide; *SO_2_*, sulfur dioxide; *PM_2.5_*, fine particulate matter with aerodynamic diameter ≤2.5 μm; *PM_10_*, particulate matter with aerodynamic diameter ≤10 μm; *O_3_*, ozone; Temp, temperature; RH, relative humidity

# Table S2. Eexcess risks (ER%) and 95% confidence intervals (95% CI) for childhood bronchopneumonia hospitalization associated with a 5 μg/m^3^ increase in NO_2_ and SO_2_ at different lag days and moving averages.

| Lag | NO2 |  | SO2 |
| --- | --- | --- | --- |
|  | ER (95% CI) |  | ER (95% CI) |
| Lag 0 | 1.71 (0.57, 2.87) |  | 3.20 (-1.12, 7.6) |
| Lag 1 | 2.73 (1.54, 3.93) |  | 5.69 (1.57, 9.98) |
| Lag 2 | 2.04 (0.96, 3.13) |  | 5.97 (2.01, 10.08) |
| Lag 3 | 2.54 (1.52, 3.58) |  | 7.59 (3.69, 11.64) |
| Lag 4 | 2.74 (1.73, 3.75) |  | 8.81 (4.94, 12.82) |
| Lag 5 | 1.75 (0.76, 2.76) |  | 5.70 (1.97, 9.57) |
| Lag 6 | 1.47 (0.48, 2.46) |  | 3.73 (0.09, 7.50) |
| Lag 7 | 2.35 (1.37, 3.35) |  | 5.47 (1.80, 9.27) |
| Lag 01 | 2.92 (1.57, 4.28) |  | 5.74 (0.99, 10.73) |
| Lag 02 | 3.64 (2.15, 5.15) |  | 7.76 (2.61, 13.17) |
| Lag 03 | 4.59 (3.00, 6.21) |  | 10.36 (4.80, 16.21) |
| Lag 04 | 5.50 (3.82, 7.21) |  | 13.26 (7.29, 19.55) |
| Lag 05 | 5.85 (4.10, 7.63) |  | 14.72 (8.40, 21.40) |
| Lag 06 | 6.12 (4.29, 7.98) |  | 15.48 (8.84, 22.52) |
| Lag 07 | 6.83 (4.93, 8.77) |  | 17.21 (10.18, 24.70) |

Abbreviations: *ER*, excess risk; 95%*CI,* 95%confidence intervals. *NO_2_*, nitrogen dioxide; *SO_2_*, sulfur dioxide.

# Table S3. Age‑stratified excess risks (ER%) and 95% confidence intervals (95% CI) for childhood bronchopneumonia hospitalization associated with a 5 μg/m^3^ increase in NO_2_ and SO_2_

| Lag | Subgroup | NO_2_ |  | SO_2_ |
| --- | --- | --- | --- | --- |
|  |  | ER (95% CI) |  | ER (95% CI) |
| Lag 0 | ≥ 6 years | 2.69 (0.65, 4.77) |  | 3.97 (-3.63, 12.16) |
|  | < 6 years | 1.29 (0.01, 2.60) |  | 2.86 (-1.99, 7.95) |
| Lag 1 | ≥ 6 years | 4.03 (1.89, 6.22) |  | 7.05 (-0.30, 14.95) |
|  | < 6 years | 2.17 (0.84, 3.53) |  | 5.20 (0.58, 10.04) |
| Lag 2 | ≥ 6 years | 3.49 (1.56, 5.45) |  | 9.74 (2.51, 17.49) |
|  | < 6 years | 1.41 (0.19, 2.65) |  | 4.46 (0.07, 9.04) |
| Lag 3 | ≥ 6 years | 3.30 (1.49, 5.15) |  | 10.40 (3.33, 17.96) |
|  | < 6 years | 2.19 (1.03, 3.36) |  | 6.43 (2.08, 10.96) |
| Lag 4 | ≥ 6 years | 3.95 (2.17, 5.77) |  | 15.85 (8.58, 23.61) |
|  | < 6 years | 2.17 (1.03, 3.32) |  | 5.97 (1.72, 10.39) |
| Lag 5 | ≥ 6 years | 3.64 (1.87, 5.45) |  | 13.79 (6.70, 21.36) |
|  | < 6 years | 0.89 (-0.24, 2.02) |  | 2.46 (-1.62, 6.71) |
| Lag 6 | ≥ 6 years | 3.54 (1.78, 5.34) |  | 8.80 (2.04, 16.00) |
|  | < 6 years | 0.52 (-0.60, 1.65) |  | 1.60 (-2.41, 5.78) |
| Lag 7 | ≥ 6 years | 4.64 (2.88, 6.43) |  | 10.36 (3.57, 17.61) |
|  | < 6 years | 1.34 (0.23, 2.47) |  | 3.41 (-0.64, 7.63) |
| Lag 01 | ≥ 6 years | 4.50 (2.07, 7.00) |  | 7.20 (-1.23, 16.34) |
|  | < 6 years | 2.26 (0.75, 3.79) |  | 5.17 (-0.17, 10.79) |
| Lag 02 | ≥ 6 years | 5.96 (3.24, 8.75) |  | 11.12 (1.83, 21.24) |
|  | < 6 years | 2.68 (1.02, 4.38) |  | 6.41 (0.67, 12.47) |
| Lag 03 | ≥ 6 years | 7.14 (4.22, 10.14) |  | 14.73 (4.63, 25.80) |
|  | < 6 years | 3.54 (1.75, 5.35) |  | 8.58 (2.42, 15.12) |
| Lag 04 | ≥ 6 years | 8.56 (5.47, 11.74) |  | 20.54 (9.44, 32.77) |
|  | < 6 years | 4.23 (2.36, 6.14) |  | 10.32 (3.77, 17.29) |
| Lag 05 | ≥ 6 years | 9.73 (6.47, 13.09) |  | 25.21 (13.14, 38.55) |
|  | < 6 years | 4.25 (2.31, 6.24) |  | 10.58 (3.71, 17.90) |
| Lag 06 | ≥ 6 years | 10.87 (7.44, 14.41) |  | 27.85 (14.97, 42.19) |
|  | < 6 years | 4.18 (2.16, 6.24) |  | 10.63 (3.46, 18.30) |
| Lag 07 | ≥ 6 years | 12.53 (8.92, 16.25) |  | 31.66 (17.78, 47.17) |
|  | < 6 years | 4.53 (2.43, 6.68) |  | 11.63 (4.08, 19.71) |

Abbreviations: *ER*, excess risk; 95%*CI,* 95%confidence intervals. *NO_2_*, nitrogen dioxide; *SO_2_*, sulfur dioxide.

# Table S4. Sex‑stratified excess risks (ER%) and 95% CI for childhood bronchopneumonia hospitalization associated with a 5 μg/m³ increase in NO_2_ and SO_2_

| Lag | Subgroup | NO_2_ |  | SO_2_ |
| --- | --- | --- | --- | --- |
|  |  | ER (95% CI) |  | ER (95% CI) |
| Lag 0 | Male | 1.61 (0.21, 3.03) |  | 2.50 (-2.74, 8.01) |
|  | Female | 1.87 (0.17, 3.60) |  | 4.28 (-2.15, 11.14) |
| Lag 1 | Male | 3.04 (1.58, 4.53) |  | 6.09 (1.04, 11.39) |
|  | Female | 2.24 (0.48, 4.03) |  | 5.04 (-1.04, 11.50) |
| Lag 2 | Male | 2.08 (0.75, 3.43) |  | 5.92 (1.10, 10.97) |
|  | Female | 1.97 (0.36, 3.61) |  | 6.00 (0.12, 12.22) |
| Lag 3 | Male | 2.68 (1.42, 3.95) |  | 7.72 (2.95, 12.70) |
|  | Female | 2.34 (0.82, 3.89) |  | 7.36 (1.58, 13.48) |
| Lag 4 | Male | 2.98 (1.75, 4.23) |  | 10.24 (5.46, 15.23) |
|  | Female | 2.37 (0.87, 3.88) |  | 6.58 (0.93, 12.54) |
| Lag 5 | Male | 1.95 (0.73, 3.19) |  | 6.92 (2.32, 11.73) |
|  | Female | 1.45 (-0.03, 2.95) |  | 3.79 (-1.67, 9.56) |
| Lag 6 | Male | 1.11 (-0.10, 2.33) |  | 3.21 (-1.20, 7.83) |
|  | Female | 2.03 (0.55, 3.53) |  | 4.51 (-0.94, 10.26) |
| Lag 7 | Male | 2.35 (1.14, 3.58) |  | 5.99 (1.49, 10.68) |
|  | Female | 2.36 (0.89, 3.86) |  | 4.58 (-0.85, 10.30) |
| Lag 01 | Male | 3.04 (1.39, 4.72) |  | 5.52 (-0.28, 11.66) |
|  | Female | 2.73 (0.74, 4.77) |  | 6.08 (-0.97, 13.63) |
| Lag 02 | Male | 3.76 (1.93, 5.63) |  | 7.52 (1.25, 14.18) |
|  | Female | 3.45 (1.24, 5.71) |  | 8.11 (0.47, 16.33) |
| Lag 03 | Male | 4.78 (2.82, 6.78) |  | 10.19 (3.43, 17.40) |
|  | Female | 4.31 (1.95, 6.73) |  | 10.58 (2.35, 19.48) |
| Lag 04 | Male | 5.80 (3.73, 7.90) |  | 13.74 (6.44, 21.53) |
|  | Female | 5.06 (2.58, 7.61) |  | 12.48 (3.71, 22.00) |
| Lag 05 | Male | 6.21 (4.06, 8.42) |  | 15.66 (7.91, 23.97) |
|  | Female | 5.31 (2.72, 7.97) |  | 13.24 (4.01, 23.29) |
| Lag 06 | Male | 6.28 (4.03, 8.58) |  | 16.08 (7.95, 24.83) |
|  | Female | 5.89 (3.18, 8.67) |  | 14.51 (4.77, 25.15) |
| Lag 07 | Male | 6.98 (4.64, 9.38) |  | 17.99 (9.37, 27.29) |
|  | Female | 6.62 (3.79, 9.53) |  | 15.96 (5.67, 27.24) |

Abbreviations: *ER*, excess risk; 95%*CI,* 95%confidence intervals. *NO_2_*, nitrogen dioxide; *SO_2_*, sulfur dioxide.

# Table S5. Excess Risks (ER) and 95% Confidence Intervals (CI) for NO_2_ and SO_2_ exposure across different lag structures, stratified by study period.

| Pollution | Lag | Full period |  | Excluding 2020–2022 |
| --- | --- | --- | --- | --- |
|  |  | ER (95% CI) |  | ER (95% CI) |
| NO2 |  |  |  |  |
|  | Lag1 | 2.73 (1.54, 3.93) |  | 2.16 (0.85, 3.49) |
|  | Lag02 | 3.64 (2.15, 5.15) |  | 2.55 (0.86, 4.26) |
|  | Lag07 | 6.83 (4.92, 8.77) |  | 4.58 (2.38, 6.82) |
| SO2 |  |  |  |  |
|  | Lag1 | 5.69 (1.57, 9.98) |  | 4.28 (0.05, 8.68) |
|  | Lag02 | 7.76 (2.61, 13.17) |  | 5.24 (-0.09, 10.86) |
|  | Lag07 | 17.21 (10.18, 24.69) |  | 10.78 (3.51, 18.57) |

Lag1, Lag02, and Lag07 indicate single-day lag (1 day prior), 0-2 day moving average, and 0-7 day moving average, respectively. Full period refers to the main analysis using all available data; Excluding 2020-2022 refers to the sensitivity analysis that omits data from 2020 to 2022 to test the robustness of the estimates and to control for potential confounding due to the COVID-19 pandemic period.

Abbreviations: *ER*, excess risk; 95%*CI,* 95%confidence intervals. *NO_2_*, nitrogen dioxide; *SO_2_*, sulfur dioxide.


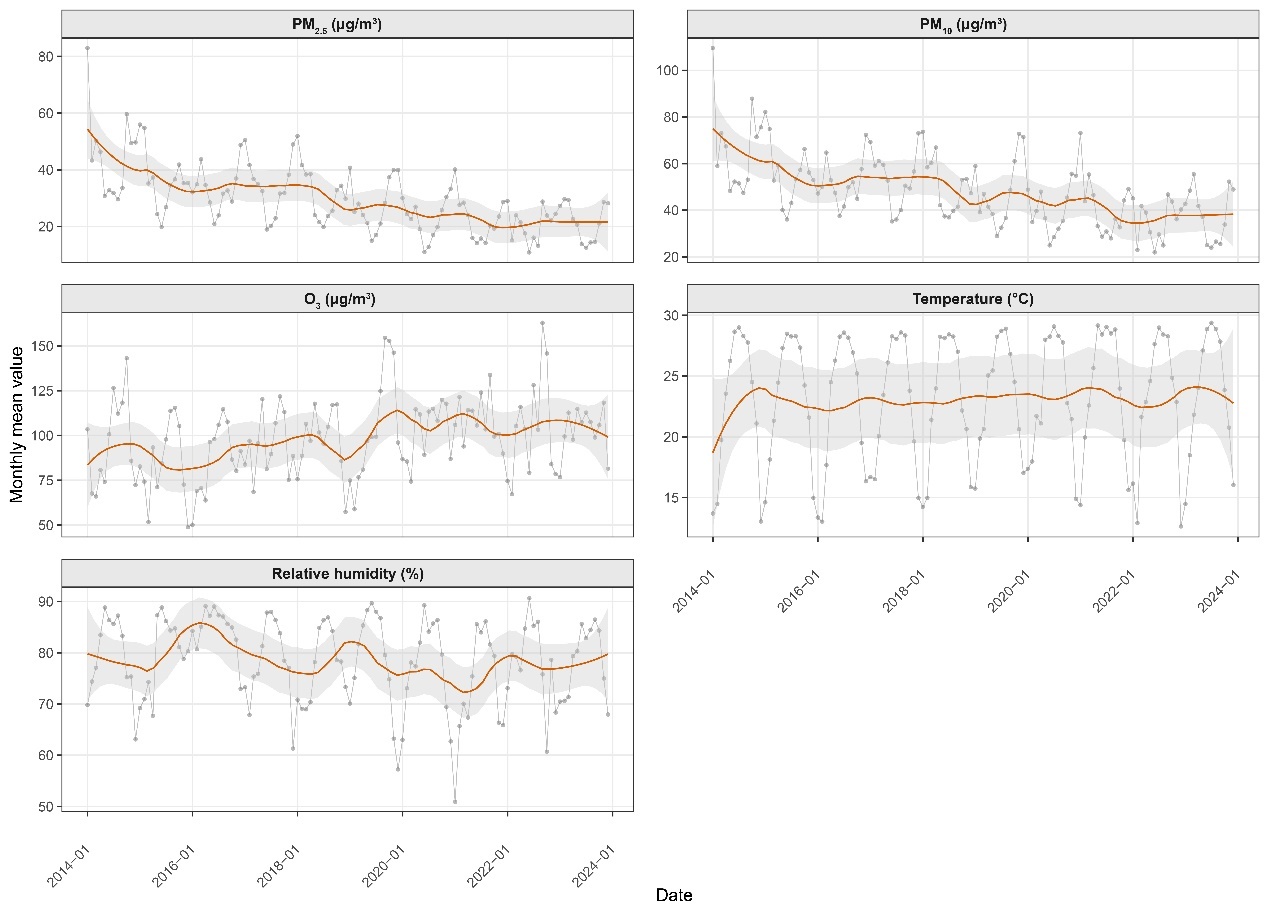


# Figure S1. Annual January trends of air pollutant concentrations and meteorological parameters from 2014 to 2023.

Gray dots and thin lines represent monthly means; the orange solid curve indicates the locally weighted regression-smoothed trend; and the gray shaded ribbon denotes the 95% confidence interval. Each panel has an independent y-axis scale, and the x-axis indicates the year of monthly aggregated data.

Abbreviations: *PM_2.5_*, fine particulate matter with aerodynamic diameter ≤2.5 μm; *PM_10_*, particulate matter with aerodynamic diameter ≤10 μm; *O_3_*, ozone.


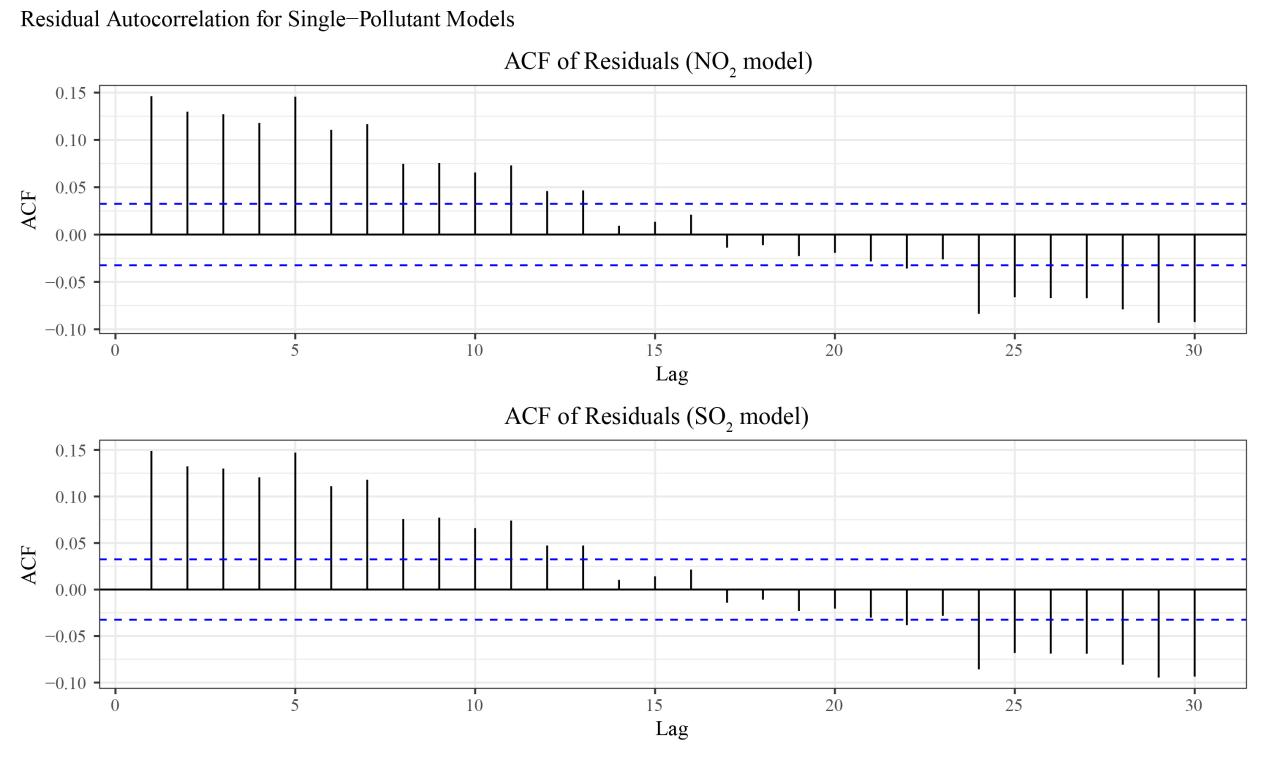


# Figure S2. Autocorrelation function (ACF) plots of deviance residuals from the single-pollutant GAM models.

(A) NO_2_ model; (B) SO_2_ model. The x-axis "Lag" represents the number of days between observations (e.g., Lag 1 = one day prior, Lag 7 = seven days prior). The horizontal dashed blue lines indicate the 95% confidence intervals. All autocorrelation coefficients generally fall within the bounds, indicating that the residuals are approximately independent with no significant serial correlation.


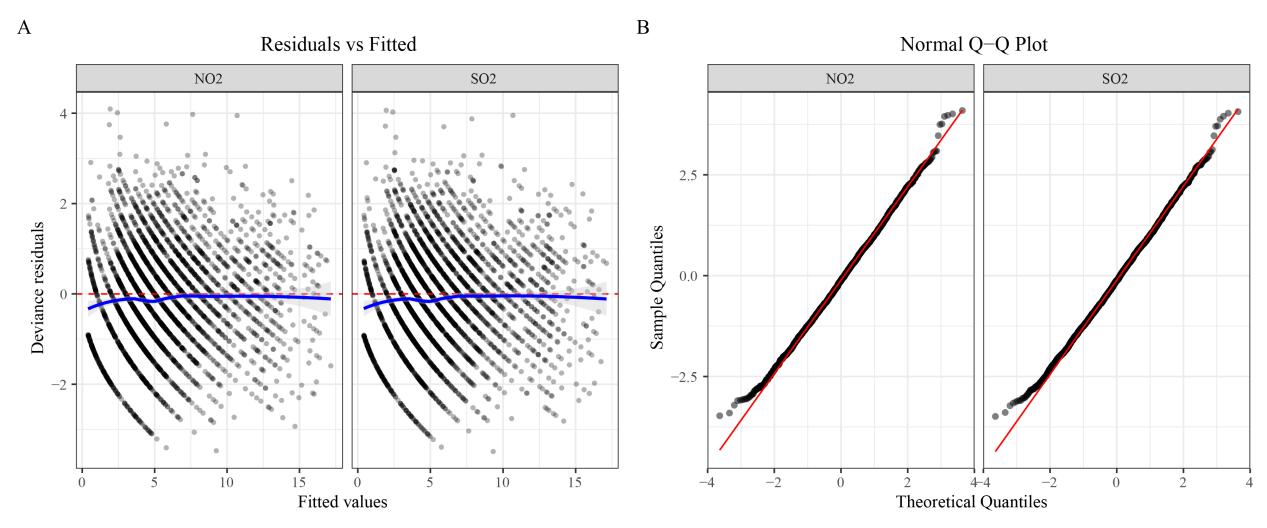


# Figure S3. Residual diagnostic plots for the single-pollutant GAM models.

1. scatterplots of deviance residuals versus fitted values, with a loess smooth (blue line) to assess homoscedasticity and systematic deviations. (B) Quantile-quantile (Q-Q) plots of residuals against theoretical normal quantiles, with the red reference diagonal line. The residuals are symmetrically distributed around zero without obvious heteroscedasticity or severe departure from normality, supporting the adequacy of the model fit.
